# Three Novel *EPCAM* Variants Causing Tufting Enteropathy in Three Families

**DOI:** 10.3390/children8060503

**Published:** 2021-06-14

**Authors:** Hasret Ayyıldız Civan, Coleen Leitner, Iris Östreicher, Anna-Maria Schneider, Malte Cremer, Johannes A. Mayr, Rainer Rossi, Thomas Müller, Andreas R. Janecke

**Affiliations:** 1 Department of Pediatric Gastroenterology, Hepatology and Nutrititon, Health Science University, Istanbul Bakırkoy Dr. Sadi Konuk Education and Research Hospital, 34147 Istanbul, Turkey; hasretayyildiz@yahoo.com; 2 Department of Pediatrics I, Medical University of Innsbruck, 6020 Innsbruck, Austria; coleen.leitner@student.i-med.ac.at (C.L.); thomas.mueller@tirol-kliniken.at (T.M.); 3 Department of Pediatrics, Klinikum Neukoelln, 12351 Berlin, Germany; iris.oestreicher@vivantes.de (I.Ö.); malte.cremer@vivantes.de (M.C.); rainer.rossi@vivantes.de (R.R.); 4 Department of Pediatrics, Paracelsus Medical University Salzburg, 5020 Salzburg, Austria; an.schneider@salk.at (A.-M.S.); h.mayr@salk.at (J.A.M.); 5 Department of Neonatology, Charité University Medical Center, 10117 Berlin, Germany; 6 Division of Human Genetics, Medical University of Innsbruck, 6020 Innsbruck, Austria

**Keywords:** tufting enteropathy, EPCAM, mutation, novel mutations, parental nutrition, intra-familial variability

## Abstract

Tufting enteropathy (TE) is caused by recessive *EPCAM* mutations, and is characterized by intractable diarrhea of congenital onset and disorganization of enterocytes. TE generally requires parenteral nutrition (PN) during childhood or intestinal bowel transplantation. We report three unrelated families with six children with TE. We highlight the high rate of disease-related mortality. We observe adequate weight gain with PN, but low to normal and stunted body length, supporting the recent notion that a short stature might be intrinsic to TE. The diagnosis of TE in the index patients from each family was delayed for months to years, even when clinical data, duodenal biopsies, or exome sequencing data were obtained early on. We identified three novel pathogenic EPCAM variants: a deletion of exon 1 that removes the ATG initiation codon, a missense variant c.326A > G (p.Gln109Arg), and nonsense mutation c.429G > A (p.Trp143*) in a compound heterozygous state with the Mediterranean splice site variant c.556-14A > G (Tyr186Phefs*6). Homozygosity for p.Gln109Arg was associated with absent EPCAM staining, and compound heterozygosity for p.Trp143*/Tyr186Phefs*6 was associated with reduced EPCAM staining in duodenal biopsies; such observations might contribute to a genotype–phenotype correlation in larger cohorts of TE patients. This study extends the clinical and molecular spectrum of TE.

## 1. Introduction

Tufting enteropathy (TE) is a rare inherited enteropathy (Mendelian Inheritance in Man #613217) presenting with intractable watery diarrhea and impaired growth in infancy. Histologically, it is characterized by disorganization of surface epithelium with focal crowding, resembling tufts, villous atrophy without mononuclear cell infiltration, and basement membrane abnormalities [1,2]. TE shows intra- and interfamilial variation in clinical severity; however, patients generally require parenteral nutrition (PN) for several years, most often throughout childhood, to avoid dehydration and to provide adequate growth [3,4]. The long-term course in TE and weaning from PN were reported in a few instances [5,6,7]. Prolonged PN therapy leads to complications, such as sepsis, thrombosis, liver disease, and a decreased quality of life [2]. A small bowel transplant is an alternate therapeutic option for TE; however, the three-year survival rate after transplant is around 30% [8].

TE was first described in 1994 [1], and less than 100 patients with molecularly confirmed etiology were reported [9]. Its prevalence is higher in countries with frequent consanguineous marriages. In some patients, punctuate keratitis, choanal atresia, and esophageal atresia may be seen as additional features, i.e., a syndromic form of TE. Mutations in the gene encoding human epithelial cell adhesion molecule (EPCAM) were first identified in TE in 2008 [8], while mutations in *SPINT2* account for patients with syndromic TE [10,11,12]. Here, we report the clinical outcomes and the identification of novel *EPCAM* variants in three unrelated families with six children affected with TE.

## 2. Patients and Methods

### 2.1. Patients

Written informed consent for molecular genetic routine investigations was obtained from the patients’ parents, and the studies were approved by the local ethics committee (votum no. AN2016-0029 359/4.5). At our centers, three families with children affected by congenital diarrhea were identified with *EPCAM* variants.

### 2.2. Molecular Investigation

We report here the results of diagnostic molecular genetic testing in patients referred with congenital intractable diarrheas. Written informed consent for genetic testing was obtained from the parents of the patients. Genomic DNA was isolated from a dried blood spot of the newborn screening card from the deceased patient 2 from family 2, and from peripheral blood samples from all other patients and parents, using an automated system (Qiasymphony, Qiagen, Hilden, Germany); however, no material for genetic testing was available from patient 2 from family 1. Whole exome target enrichment was performed with 0.5 µg of genomic DNA from the index patients in families 1 and 3, and with the Agilent SureSelectHuman All Exon 60 Mb Capture kit (Agilent Technologies, Santa Clara, CA, USA); 2 × 100 bp paired-end sequences were produced using an Illumina Hi-Seq4000 platform. Variants were called in genes known to cause forms of congenital diarrheas using the SeattleSeq Annotation server (http://snp.gs.washington.edu/SeattleSeqAnnotation138/ (accessed on 1 February 2021)), and were filtered for the autosomal recessive mode of inheritance, predicted effect on protein expression, and allele frequency of <0.005 in the Exome Aggregation Consortium (http://exac.broadinstitute.org/ (accessed on 1 February 2021)) database. The missense variant identified in family 2 was evaluated in silico for pathogenicity by PolyPhen-2 (http://genetics.bwh.harvard.edu/pph2 (accessed on 1 February 2021)), CADD (http://cadd.gs.washington.edu/score (accessed on 1 February 2021)), MutationTaster (http://www.mutationtaster.org/ (accessed on 1 February 2021)), and PROVEAN (http://provean.jcvi.org/seq_submit.php (accessed on 1 February 2021)). A copy number detection in targeted NGS data was performed using panelcn.MOPS (https://ml.jku.at/software/panelcnmops/ (accessed on 1 February 2021)). The coding region of the *EPCAM* gene was directly sequenced in the index patient from family 2 (primer sequences available from the authors upon request). Quantitative PCR (family 1) and Sanger sequencing (families 2 and 3) were used to determine the segregation of identified *EPCAM* variants in the families. *EPCAM* variant designations are based on the NCBI reference sequence NM_002354.3.

## 3. Results

### 3.1. Clinical Findings

**Figure 1:**
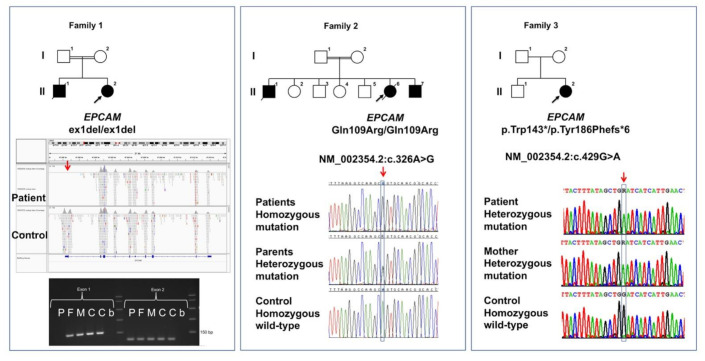
Three novel families with TE and novel *EPCAM* mutations.

Clinical details of all six patients from three families with novel EPCAM variants are compiled in Table 1, and the family trees are shown in Figure 1.

In the index patient from family 3, a second esophagogastroduodenoscopy was performed at the age of six months. While characteristic histopathological changes for TE were not encountered, immunohistochemistry revealed nearly absent *EPCAM* expression (with antibody Ber-EP4), suggesting a diagnosis of TE. At the age of eight months, exome sequencing revealed compound heterozygous *EPCAM* variants, resulting in a diagnosis of TE.

While weight gain appeared sufficient with PN or partial PN in these patients, body length was still mildly to moderately affected by the disease. Prolonged hyperbilirubinemia and cholestasis were seen in two out of six patients before PN initiation.

Of note, three out of six patients had died at the ages of 3.5, seven, and eight years, respectively, from catheter-related complications, emphasizing the severity of this disorder. Early in the disease course of each patient, stool examinations for infectious agents, abdominal ultrasound, and testing for cystic fibrosis were without pathological findings, and fecal calprotectin concentrations were normal.

Of note, the diagnosis of TE in the index patients from all three families was delayed. An autosomal recessive form of diarrhea was suspected in the index patient from family 1 at age one year, and research exome sequencing rendered a definite diagnosis of TE at the age of two years. The parents had denied a gastroduodenoscopy in their children.

Family 2 had six children, of whom three were affected by TE (Figure 1); the first child of the family had suffered from intractable diarrhea from birth, and died at age eight years from catheter-related septicemia. Duodenal biopsies taken at the ages of two and four years showed subtotal to total villus atrophy and mild inflammation, whereas postmortem re-evaluation of the duodenal biopsies from this patient revealed occasional tufting of villus enterocytes and a lack of *EPCAM* staining. This result prompted targeted molecular testing of *EPCAM*.

### 3.2. Molecular Findings

In family 1, a novel homozygous deletion of exon 1 and flanking sequences of *EPCAM* (NM_002354.3):c.1_46del (p.?) were identified in patient 1 by exome sequencing (Figure 1). The exon 1 deletion localized within a 47-Mb region of homozygosity on chromosome 2p21 in this patient, leading to the hypothesis of identity by descent of the disease-causing deletion due to remote parental consanguinity. The deletion removes the translation initiation codon, and likely represents a “null mutation.” Quantitative PCR showed that both parents were heterozygous for this deletion; material for genetic testing was not available from the first affected child.

In family 2, direct *EPCAM* Sanger sequencing identified a novel homozygous c.326A > G (p.Gln109Arg) variant in all three affected individuals. Both parents were heterozygous carriers of this variant. This variant affects the function of *EPCAM* as predicted by three of four employed in silico programs (PROVEAN score: −3.521; CADD phred score: 27.1; MutationTaster score: 0.999), whereas Polyphen-2 considers the variant as benign (HumVar score: 0.146). This variant is not listed in the ExAC and gnomAD databases, indicating that it is not a frequent polymorphism.

The proband from family 3 is a compound heterozygote for two *EPCAM* variants: the *EPCAM* variant c.429G > A (p.Trp143*) is listed as pathogenic in the ClinVar database (https://www.ncbi.nlm.nih.gov/clinvar/variation/239127/#id_third (accessed on 1 February 2021)); however, neither clinical details nor zygosity are provided with the variant, and therefore, we labelled it as a novel variant. It was found in-trans with the well-studied *EPCAM* variant c.556-14A > G (p.Tyr186Phefs*6). Both variants lead to premature stop codons.

Simplified family trees of families 1–3 as described in case reports are shown. Circles denote female and squares denote male individuals; filled symbols denote individuals affected by TE. Five out of six affected individuals were shown to harbor bi-allelic *EPCAM* variants, and all parents were heterozygous carriers of variants. Only novel *EPCAM* variants are shown under each pedigree. In family 1, the deletion of *EPCAM* exon 1 as compared with a control sample is shown in exome sequencing data visualized with the integrative genome viewer (IGV) software (panel under the pedigree drawing). The lowest panel shows an electrophoresis picture of semi-quantitative PCRs for *EPCAM* exons 1 and 2. No PCR product was obtained with the patient’s DNA sample (P), and parents (F, M) showed decreased intensities as compared with two controls (C, C) for exon 1 in contrast to equal intensities observed for exon 2 amplification for all individuals; b, blank (no template control).

## 4. Discussion

*EPCAM* deficiency causes TE by intestinal barrier disruption through the loss of *EPCAM*-mediated cell–cell interactions at the basolateral membrane of the intestinal epithelium [13,14]. In addition, the interaction of *EPCAM* with claudin-7 and the actin cytoskeleton to modulate tight junctions might be disrupted [15], and alterations in cell differentiation and enterocyte function were described in TE, likely contributing to the development of the clinical symptoms [4,16]. TE patients who do not harbor *EPCAM* mutations present with TE and a pattern of congenital malformations, a syndromic form of TE caused by mutations in *SPINT2* (MIM# 605124) [3,11,12]. *SPINT2* encodes a serine protease that might be involved in regulating EPCAM and claudin-7 availability at the plasma membrane [17].

To date, pathogenic *EPCAM* variants were identified in about 100 individuals with TE [7,9,18], and most *EPCAM* variants were rare or confined to single families. There are seven out of 42 variants that have been identified in more than two TE patients each [7,9]. Our identification of three novel *EPCAM* variants in three families extends this genetic heterogeneity further. The index patient in family 3 carries the novel premature stop codon in-trans with the second most frequent variant, the pathogenic Mediterranean *EPCAM* variant c.556-14A > G (p.Tyr186Phefs*6), that has been shown to result in defective RNA splicing and loss of protein [3,9]. The deletion of exon 1 in family 1 is expected to abolish the production of a functional protein as the translation start codon is removed. The novel missense variant p.Gln109Arg is considered pathogenic, as it replaces an evolutionary highly-conserved polar glutamine residue within an alpha-helix of *EPCAM* with a larger and charged arginine, and it is present in a homozygous state in all three affected individuals in family 2. In addition, homozygosity for the p.Gln109Arg variant was associated with absent *EPCAM* staining in intestinal epithelium.

Compound heterozygosity for p.Trp143*/Tyr186Phefs*6 was associated with nearly absent *EPCAM* staining in duodenal biopsies. A number of *EPCAM* genotypes causing TE are associated with absent intestinal EPCAM staining [19,20], and such observations might eventually contribute to a genotype–phenotype correlation in larger cohorts of TE patients [9].

Very recently, a lack of *EPCAM* staining along the lateral membrane of biliary epithelial cells was reported in three TE patients, associated with mild cholestasis and a reduction of bile ducts in a portal tract [21]. In our case series, hyperbilirubinemia and cholestasis were present in two patients, respectively, on admission at an age of six weeks and before the start of PN, potentially resulting from *EPCAM* deficiency in biliary epithelial cells.

Our clinical reports demonstrate a number of issues related to TE. First, establishing the diagnosis of this rare disease in a new patient might still take months to years.

Rare monogenic enteropathies should be considered as differential diagnoses in infants presenting with congenital diarrhea, histopathology should comprise *EPCAM* immunohistochemistry, as the characteristic morphological changes of TE are sometimes difficult to encounter, and genetic testing needs to consider the occurrence of pathogenic and intronic *EPCAM* variants.

We suggest to perform exome sequencing—clinical or whole exome sequencing—within the first four weeks in any patient with protracted diarrhea. Sequencing costs have dropped considerably recently, and genetic testing is less invasive than performing a gastroduodenoscopy in a young and ill patient. Exome sequencing needs a minute amount of blood for DNA extraction, and can target > 100 genes that cause isolated and syndromic forms of congenital diarrheas and immunodeficiencies. In addition, identifying the characteristic hallmarks of TE by histopathology can be challenging, and the diagnosis might be overlooked.

Our reports demonstrate the need to diagnose and treat PN-related complications to decrease the mortality of this disorder. We confirm previous observations that patients with TE can survive the first year(s) of life with breastfeeding or formula, despite the severe diarrhea, at the cost of stunted growth [5,22].

A few long-term observations suggest that enteral nutrition is gradually better tolerated in TE, and weaning from PN can be eventually achieved [6,7]. However, based on the largest clinical patient series (n = 18) to date, it has been suggested that the observed stunted growth in all patients might be intrinsically related to this disorder, and might not be overcome with PN [7]. So far, this hypothesis is supported by our patient series. In this respect, it will be of great interest to follow-up with the three patients described here who were diagnosed and treated relatively early in the first two years of life. Our study extends the clinical and molecular spectrum of TE.

## Figures and Tables

**Table 1:** Clinical findings in this study.

	Family 1		Family 2			Family 3
	Patient 1	Patient 2	Patient 1	Patient 2	Patient 3	Patient 1
Parental consanguinity	+	+	+	+	+	-
Pregnancy and gestational age	Preeclampsia 34 weeks	Born at term	Born at term	n.a.	38 weeks	41 weeks
Birth measures	W = 2645 g (z = 0.58), L = 48 cm (z = 0.52) HC = 31.3 cm (z = −0.57).	L = 53 cm (z = 0.61), HC = 35 cm (z = 0.04)	W = 3415 g (z = 0.23), L = 51 cm (z = −0.05), HC = 34 cm (z = −0.20)	n.a.	W = 3700 g (z = 0.61), L = 56 cm (z = 1.86), HC = 37 cm (z = 1.92)	W = 3660 g (z = −0.14), L = 52 cm (z = −0.44), HC = 34 (z = −0.90)
Onset of persistent diarrhea	First day of life	**One year**	Six weeks of life	First day of life	Six weeks of life	Second month of life
Disease course	Intractable diarrhea and failure to thrive, weighing 5710 g (z = −3.63) at one year. Breast milk feeding in the first year of life. No duodenal biopsy was obtained.	Intractable diarrhea and failure to thrive from one year of age. No duodenal biopsy was obtained.	Intractable diarrhea and failure to thrive. **TBIL of 3.47 mg/dl (expected 0.1–1.20), direct bilirubin (DBIL) 3.01 mg/dl (0.0–0.3)** prior to PN initiation.	Intractable diarrhea and failure to thrive. PN and PEG tube feeding. Duodenal biopsies showed total villus atrophy and mild inflammation, and findings of TE on re-evaluation post-mortem. Birth of patient 1 to this family led to a diagnosis of TE.	Intractable diarrhea and failure to thrive. Fully breastfed for six weeks. **Hyperbilirubinemia (TBIL = 8.75 mg/dl, DBIL = 0.56 mg/dl)** prior to PN initiation.	Intractable diarrhea and severe failure to thrive weighing 3910 g (z −2.87) at three months with infant formula. Stool pH low (pH = 6.0), steatorrhea. Initial suspicion of cow’s milk protein intolerance. Esophagogastroduodenoscopy and colonoscopy at four months of age excluded inflammatory bowel disease; gastroduodenoscopy at six months revealed nearly absent *EPCAM* expression.
Age at definite diagnosis of TE/method	Two years/exome sequencing	Post-mortem, in retrospect	Three months/*EPCAM* sequencing	Post-mortem, in retrospect/immunohistochemistry	Nine weeks of age/*EPCAM* sequencing	Eight months/immunohistochemistry and exome sequencing
Age at last examination or death	Three years	Died age seven years	Died age 3.5 years from multi-organ failure	Died age eight years from catheter-related septicemia and multiple organ failure	Three years	Two years
Body measuresat last examination	W = 12.5 kg (z = −0.96) **L of 82 cm (z = −3.4)** HC = 49 cm (z = −0.34)	W = 7.5 kg (z = −15.86).	W = 9.4 kg (z = −2.97), **L = 81 cm (z = −3.22).**	n.a.	W = 15 kg (z = 0.08), **L = 92 cm (z = −1.47),** HC = 50.7 cm (−0.05)	W = 10.45 kg (z = −1.23), **L = 77.5 cm (z = −2.74)**, HC = 49 cm (z = −0.25)
Current treatment	≈90% of calories and fluid by central venous catheter; pancreatic enzymes orally, at home.	n.a.	n.a.	n.a.	Family food (1277 kcal/day), PN for 12 h/day (800 mL, glucose = 12 g/kg, protein = 23.5 g/kg, lipids = 2 g/kg, respectively, 80 kcal/kg), PN paused every fourth day	Enteral nutrition: Solid low-protein foods (220 kcal/d and 8 g of protein/day), 550 mL Basic-p 17% + 15 mL Liquigen/d, approximately 578 kcal/d). PN for 12 h/day (glucose = 2.5 g/kg, amino acids = 2.9 g/kg, lipids = 0.8 g/kg, corresponding to 283 kcal/d)
Current status	Persistent diarrhea. Psychomotor development is age-appropriate.				Formed to mucous stools 1–3 times/day, abdomen extended with weakened peristalsis. Age-appropriate psychomotor development.	Formed to mucous stools 2–3 times/day, abdomen distended but soft, no palpable resistance. Age-appropriate psychomotor development.
Additional findings	AST elevation (46.5 U/L (0–32)) with PN.	Ventriculostomy for aqueduct stenosis. Marked scoliosis.				Persisting mild acidosis (pH = 7.31), mild AST, and ALT elevation.

L, length; W, weight; HC, head circumference; n.a., not available; N.A., not applicable; PN, parenteral nutrition; bold font indicates findings elaborated in the text.

## Data Availability

The data presented in this study are available on reasonable request from the corresponding author.
